# A Review of the Potential of Virtual Walking Techniques for Gait Rehabilitation

**DOI:** 10.3389/fnhum.2021.717291

**Published:** 2021-11-03

**Authors:** Omar Janeh, Frank Steinicke

**Affiliations:** ^1^Department of Computer Engineering, University of Technology, Baghdad, Iraq; ^2^Human-Computer Interaction, Department of Informatics, Universität Hamburg, Hamburg, Germany

**Keywords:** virtual locomotion techniques, virtual reality, gait disorders, therapeutic advances, rehabilitation

## Abstract

Virtual reality (VR) technology has emerged as a promising tool for studying and rehabilitating gait disturbances in different cohorts of patients (such as Parkinson's disease, post-stroke, or other neurological disorders) as it allows patients to be engaged in an immersive and artificial environment, which can be designed to address the particular needs of each individual. This review demonstrates the state of the art in applications of virtual walking techniques and related technologies for gait therapy and rehabilitation of people with movement disorders makes recommendations for future research and discusses the use of VR in the clinic. However, the potential for using these techniques in gait rehabilitation is to provide a more personalized approach by simulate the experience of natural walking, while patients with neurological disorders are maintained localized in the real world. The goal of our work is to investigate how the human nervous system controls movement in health and neurodegenerative disease.

## 1. Introduction

The emergence of virtual reality (VR) as a therapeutic tool has provided important insights for developing potential movement therapies for patients with neurological conditions, such as Parkinson's disease (Lei et al., 2019), stroke (Huygelier et al., 2021) or other nervous system iseases (Liu et al., 2018). Although, its research in rehabilitation is becoming more widespread as technology becomes more accessible and affordable, the utilization of VR is not yet regularly used in clinical rehabilitation settings. However, VR provides a novel platform for the development of unique and customizable interventions, which enables new interventions by manipulating training duration or intensity as well as multi-sensory feedback to satisfy clinical demands for intensive and repetitive patients training (Deutsch and Mirelman, 2007; Kiefer et al., 2013), and increase their interest in the rehabilitation process by letting patients experience immersion [e.g., using head mounted displays (HMD)] or non-immersion (e.g., using 2D displays with a limited field of view) virtual environments (VEs), so that patients' treatment compliance is effectively improved (Peñasco-Martín et al., 2010; Gallagher et al., 2016). Thus, more immersive displays have a higher opportunity to present a fully artificial digital environment that results in a high sense of presence (Milgram and Kishino, 1994). Slater (2003) has defined presence as the feeling of being in an environment even when the person is not physically present and leading to behavior that resembles the subject's situation in the environment.

Rehabilitation interventions in VEs can manipulate practice conditions to engage motivation, motor control, cognitive processes and sensory feedback-based learning mechanisms (Levin et al., 2015). Porras et al. (2018) suggested that implementation of patient-tailored motor learning strategies into the design and planning of VR interventions may enhance the efficiency and improve the therapeutic outcome. To this end, the general principles of motor learning can be well applied and integrated in VR training by providing goal-oriented, repetitive and varied practice that is adjusted to the abilities of the user (Deutsch and Mirelman, 2007; Langhorne et al., 2011). Therefore, when developing VR interventions, it is important to consider both the construction of the VE and the interfaces for measurement and feedback that accompany them (Weiss et al., 2006). Together, novel forms of therapeutic interventions can be used to evaluate and treat specific aspects of the human gait (Martens et al., 2017). Recent research has increasingly focused on the use of VR in rehabilitation, including to enhance walking (Mirelman et al., 2011, 2013; de Rooij et al., 2016, 2019).

Virtual walking (i.e., based on real walking) is considered the most intuitive way of navigation in VEs and is also found to be more presence-enhancing compared to other navigation techniques (Usoh et al., 1999). Furthermore, it is proven to be superior over other techniques across users' navigational tasks (Ruddle and Lessels, 2009), cognitive map buildings (Ruddle et al., 2011), and cognitive demands (Marsh et al., 2013). Therefore, a variety of virtual walking techniques have been proposed (see section 3), including walking in place (Slater et al., 1995a,b), redirected walking (Razzaque et al., 2001) or omnidirectional treadmill (Darken et al., 1997). However, there is an increased number of recent studies that use virtual walking techniques for medical and rehabilitative purposes, which provide insights into the future of gait rehabilitation in VR. For instance, Martelli et al. (2018), Janeh et al. (2019b), and Rockstroh et al. (2020) have used a real walking technique, which allows the user to walk about the space in a controlled VE. Other researchers have used new technologies of locomotion devices, such as Strider (Freiwald et al., 2020), 360° VR video-based immersive cycling training system (Lee et al., 2021) and KatWalk omnidirectional treadmill (Cherni et al., 2021). In addition, a recent study by Cai et al. (2021) has shown that WIP is feasible on gait rehabilitation of stroke patients, which translates the viewpoint when the user marches in a stationary location.

The goal of this review is to summarize insights from studies on locomotion techniques in VEs that illuminate the role of movement variability for gait therapy and discuss options for VEs to manipulate task attributes to provide novel forms of feedback and guidance. However, it can inform clinical decision-making and future practice about how to best apply virtual walking techniques in gait rehabilitation, and identify the walking task delivery under the different interface conditions, to demonstrate that the acquired skills from VE practice can be transferred to the real world. We summarize the state of the art of virtual walking techniques for gait rehabilitation in terms of technical, perceptual, cognitive aspects, as well as simulator sickness aspects that must be components of VEs for transfer to occur.

## 2. Human Gait

Human gait refers to the repetitive locomotion pattern of how a person walks. Although, the process appears automatic and easy, gait is actually a complex and high-level motor function (Mansfield and Neumann, 2009). In order to analyze and evaluate how a person walks, it is necessary to isolate the shortest, unique, repeatable task during gait. This task is called the (bipedal) gait cycle that requires movements from the right and left sides of the body. In normal gait, the average duration of a gait cycle will be very similar for the left and right sides. In pathological (i.e., abnormal) gait, there may be a pronounced difference between the two sides, leading to arrhythmic gait patterns (Uchytil et al., 2017).

### 2.1. Phases of the Gait Cycle

**Figure 1:**
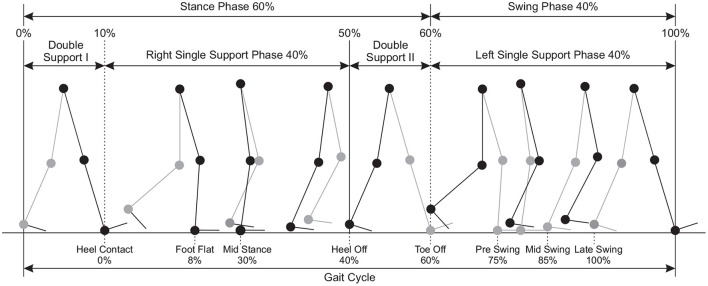
Phases of gait cycle defined for rhythmic natural walking and their proportions as percentages.

A gait cycle begins when the heel of one foot touches the ground and ends after the leg and body have advanced through space and time and the heel of that same foot hits the ground again. Realizing aspects of the gait cycle such as phasic, time, spatial and pressure measures, which can be measured an utilized to determine the quality of a person's gait. The cycle includes a period when the leg is in contact with the ground, which is followed by a period when it is advancing through space. Because of the dynamic and continuous nature of walking, the gait cycle is described as occurring between 0 and 100% (Figure 1). It can be distinguished into two primary phases: (i) the stance and (ii) swing phases, which alternate for right and left lower limbs.

- *Stance phase* describes the portion of the gait cycle when the foot is in contact with the ground, which makes up to 60% of the gait cycle. Within a stance phase, the double support represents approximately 20% and single support represents approximately 40% of the gait cycle (Inman et al., 1981). Therefore, when a foot is in a swing phase the other foot should be in a single support phase. When a foot is in a stance phase, it goes through a double support phase 10% of the initial stance phase, a single support phase 40%, and another double support phase 10% of the end of stance.
- *Double support* denotes the amount of time that a participant spends with both feet on the ground during one gait cycle.
- *Single support* describes the time elapsed between the last contact of the current footfall to the first contact of the next footfall of the same foot. It is equivalent to the swing time.
- *Swing phase* is the portion of the gait cycle when one foot is in the air. It is equivalent to the single support time of the opposite foot.

The phases of swing and stance are further divided into eight events during the gait cycle (Perry and Davids, 1992); five of which occur in the stance phase, when the foot is on the ground, and three in the swing phase, when the foot is moving forward through the air (Figure 1).

1. *Heel contact:* the heel or another part of the foot contacts the ground (at 0% of the gait cycle).
2. *Foot flat* the period that the entire plantar aspect of the foot is on the ground (at 8% of the gait cycle).
3. *Mid stance* is the point where the body weight passes directly over the supporting lower extremity (at 30% of the gait cycle).
4. *Heel off* describes the instant the heel leaves the ground (at 40% of the gait cycle).
5. *Toe off* describes the instant the toe leaves the ground (at 60% of the gait cycle).
6. *Pre swing* describes the period from toe off to mid swing (at 75% of the gait cycle).
7. *Mid swing* is the period when the foot of the swing leg passes next to the foot of the stance leg (at 85% of the gait cycle). This corresponds to the mid stance phase of the opposite lower extremity.
8. *Late swing* the period ranging from mid swing until heel contact (at 100% of the gait cycle).

### 2.2. Control of the Gait Cycle

Bipedal locomotion is accomplished through a complex and coordinated pattern of nerve signals, sent to the muscles, which in turn move the joints, the limbs and the remainder of the body (Duysens and Van de Crommert, 1998; Whittle, 2014). During walking in the real world, vestibular, proprioceptive, and efferent copy signals, as well as visual information create a consistent multi-sensory representation of a person's self-motion, i.e., acceleration, velocity and walking direction (Dietz, 2002; Takakusaki, 2013). Modifying the sensory information during the movement can come from either proprioceptive information or efference copies of the motor command during the preparation for motor output (Pynn and DeSouza, 2013). The control of locomotion involves the use of afferent information from a variety of sources in the visual, auditory, vestibular and proprioceptive systems (Dietz, 2002). Efference copies are those neural representations of motor outputs that predict reafferent sensory feedback and modulate the response of the corresponding sensory modalities. Also, accessing a copy of the efferent command allows the brain to prepare for the consequences of an intended motion before it has occurred (Harris et al., 2002).

The voluntary control of movement and high-level modulation of gait patterns is originated at the supraspinal level. The latter regulates both the central pattern generator and reflex mechanisms (Dietz, 2002). Also at the supraspinal level, information from vestibular and visual systems are incorporated, which are crucial for the maintenance of balance, orientation, and control of precise movement (Dietz, 2002). Efferent stimulation is transmitted through motor neurons to individual muscle groups, which are recruited to affect the movement. Afferent feedback, including that from proprioceptors of the muscles and joints and mechanoreceptors of the skin, is used to directly modulate motor commands via mono- and polysynaptic reflex arcs, thus contributing to the efficiency of gait under normal conditions and stability of gait in the face of unexpected perturbations (Tucker et al., 2015).

### 2.3. Gait in Older Adults

The gait of the older adults is subject to two influences (Whittle, 2014): the effects of age itself and the effects of pathological conditions, such as osteoarthritis and parkinsonism, which become more common with advancing age. The gait of the older adults appears to be simply a slowed down version of the gait of younger adults. Furthermore, the differences between the gait of the younger and the older adults are described by Murray et al. (1969), which suggested that the purpose of gait changes in the elderly is characterized by a cautious attitude of walking, which is essentially an exaggeration of the gait changes which normally occur with age. For instance, decreasing the step length and increasing the step width make it easier to maintain balance while walking. Increasing the cycle time leads to a reduction in the percentage of the gait cycle for which there is only single support, since the increase in cycle length is largely achieved by lengthening the stance phase and hence the double support time. A comprehensive review of the changes in gait with advancing age was given by Prince et al. (1997).

Given biological aspects of walking, gait performance is determined by continuous, ongoing postural adjustments by several types of control mechanisms. Stereotypical patterns of synergic muscle group activation (Diener et al., 1988) need to be scaled appropriately by peripheral sensory feedback (Diener et al., 1988) and centrally generated, anticipatory motor programs (Horak and Macpherson, 1996). It has proposed that postural alignment requires three different processes (Horak et al., 1992): (i) sensory organization and weighting of the orientation senses such as somatosensory proprioceptive, visual and vestibular information, (ii) motor adjustment processes involved with executing coordinated and properly scaled neuromuscular responses and (iii) background tone of muscles through which balance changes are compensated. The process of sensory organization seems to be hierarchically organized at different levels, these systems should be coherent, any conflicting orientation inputs must be quickly suppressed in favor of those congruent with the internal reference, otherwise postural and gait performance worsens (Massion, 1998; Mergner and Rosemeier, 1998).

## 3. Virtual Walking Techniques

As in the real world, most immersive virtual environments are usually suitable to be explored by walking. However, allowing VR users unconstrained walking requires huge free-space areas in which the movements of the user can be tracked. In particular, in a VE the space may have infinite size and the user should be able to walk and explore that space freely. However, in real physical spaces users have constrained space. If the virtual space and the real space have similar sizes, a one-to-one mapping can be used for navigation, but if the virtual space is larger than the real space, the users may eventually walk outside the real tracking space. This interrupts the tracking and may breaks in presence and lead to reduce user experience. To overcome this limitation, some techniques have been developed to enable users to explore larger VEs with real walking. In this section, we summarize some of these most fundamental approaches:

### 3.1. Walking in Place

**Figure 2:**
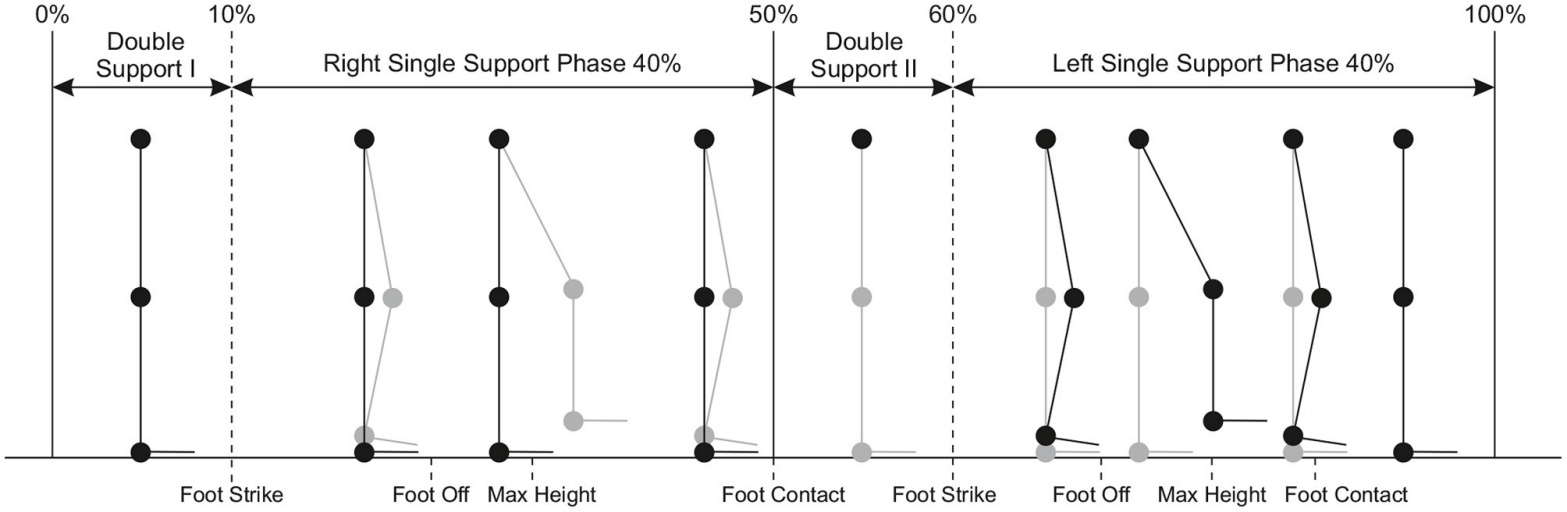
Phases of gait cycle defined for rhythmic walking in place and their proportions as percentages.

In *walking-in-place* (WIP) interfaces, users perform stepping-like movements without forward motion of the body, but a virtual forward motion is induced instead. The diagram in Figure 2 shows the gait cycle of WIP technique; the significant difference is that the single support periods of normal walking are replaced by foot off, maximum height and foot contact. In this technique, users make body gestures similar to real world walking, without actually moving with respect to the physical environment. This way, users can walk virtually and explore a larger virtual environment. Important advantages of WIP technique include: cost effectiveness (Feasel et al., 2008), naturalness (Usoh et al., 1999), stronger feeling of presence and easier to learn compared to other approaches (Slater et al., 1995b; Templeman et al., 1999), and proprioceptive feedback similar to real walking (Slater et al., 1994). However, since displacement in the real world is prevented with WIP technique, vestibular feedback as in real walking is not possible. One of the first scientific implementations of the walking in place technique was published by Slater et al. (1995b) and Slater et al. (1995a). In that work, head movements were analyzed while performing WIP gesture Figure 4A, and virtual walking was triggered by the movement of the head. The latency was substantial; the system required four steps in place to start the virtual walking, since false-positive steps (moving viewpoint when the user is not walking in place) were considered more confusing than a late start. Similarly, the system looked for no steps for two cycles to stop the virtual walking. Since then, different aspects of the walking in place technique have been examined, such as step detection, start and stop latency (Feasel et al., 2008), and smooth motion (Whitton and Peck, 2013).

Wendt et al. (2010) proposed system used a biomechanical state machine to control the virtual walking, and found more consistent output speeds compared to a study by Feasel et al. (2008). A similar study by Kim et al. (2012) have proposed a technique that triggers WIP technique using the inertial sensors embedded within two smart phones attached to the user's ankles in order to track leg movement in real time. Usually, most WIP techniques rely on gestures for walking input and control, for instance, a so-called stepping gesture, similar to soldiers marching in place. Nilsson et al. (2013a) performed a study comparing this gesture to two alternative gestural inputs: (i) a gesture where the user alternately bends each knee, thus moving the lower leg backwards, and (ii) a gesture where the user in turn taps each heel against the ground without breaking contact with the toes. Furthermore, the perceived required physical effort for the tapping gesture (**Figure 4B**) was closer to real walking. In another study by Nilsson et al. (2013b), some of those authors examined two more input gestures (i.e., hip movement and arm swinging). The results showed that arm swinging (**Figure 4C**) was perceived as natural as the original WIP technique. Moreover, Langbehn et al. (2015) have proposed WIP technique (**Figure 4D**) that involves a novel way of scaling the speed derived from the steps in place (i.e., the user is able to increase the speed by leaning the torso forward).

### 3.2. Redirected Walking

**Figure 3:**
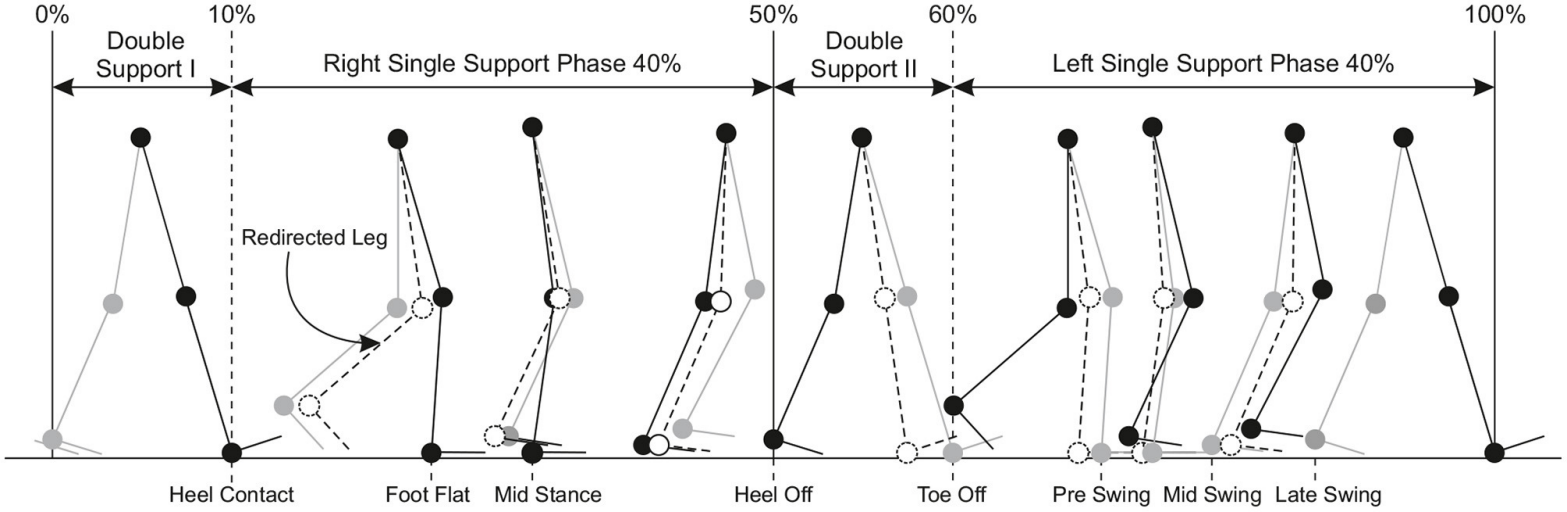
Phases of gait cycle defined for redirected walking and their proportions as percentages.

*Redirected Walking* (RDW) enables users to explore a virtual world that is considerably larger than the real world (Steinicke et al., 2009b). The idea is that users walk on different paths in the real world, which may vary from the paths they perceive in the VE (Bruder et al., 2013; Vasylevska and Kaufmann, 2017; Nilsson et al., 2018). For instance, using curvature gains the user effectively starts walking in small circles in the physical space while having the illusion of being able to walk straight in the VE (Razzaque et al., 2001). More particularly, (Figure 3) illustrates redirection of gait in a VE where the change of direction (i.e., redirected leg) is opposite to the contact leg, such as turning left while the right leg is in contact with the ground (Hase and Stein, 1999). This turning strategy is very similar to the one used in normal straight walking and tends to enlarge the step width, which minimizes the risk of falling, maximizes the possibility of fast change of directions, and ensures continuity of the walking path (Patla et al., 1999). However, redirection causes a sensory mismatch between the visual and bodily feedback elicited by the rotating VE during walking (Rothacher et al., 2018). It is found that, when only visual input is supplied, people can successfully estimate the amount of change in direction but not the path they followed (Lappe et al., 1999). This makes it possible to manipulate the visual flow to keep the users in the tracking area without being able to notice the manipulations if a physical space of at least 45*m*^2^ is available (Steinicke et al., 2009b). These experiments have been replicated with different settings and extended several times (Kopper et al., 2011; Bruder et al., 2012; Freitag et al., 2016). For instance, Grechkin et al. (2016) found that an area of approximately 25*m*^2^ can be sufficient for unlimited straight walking in a VE.

**Figure 4:**
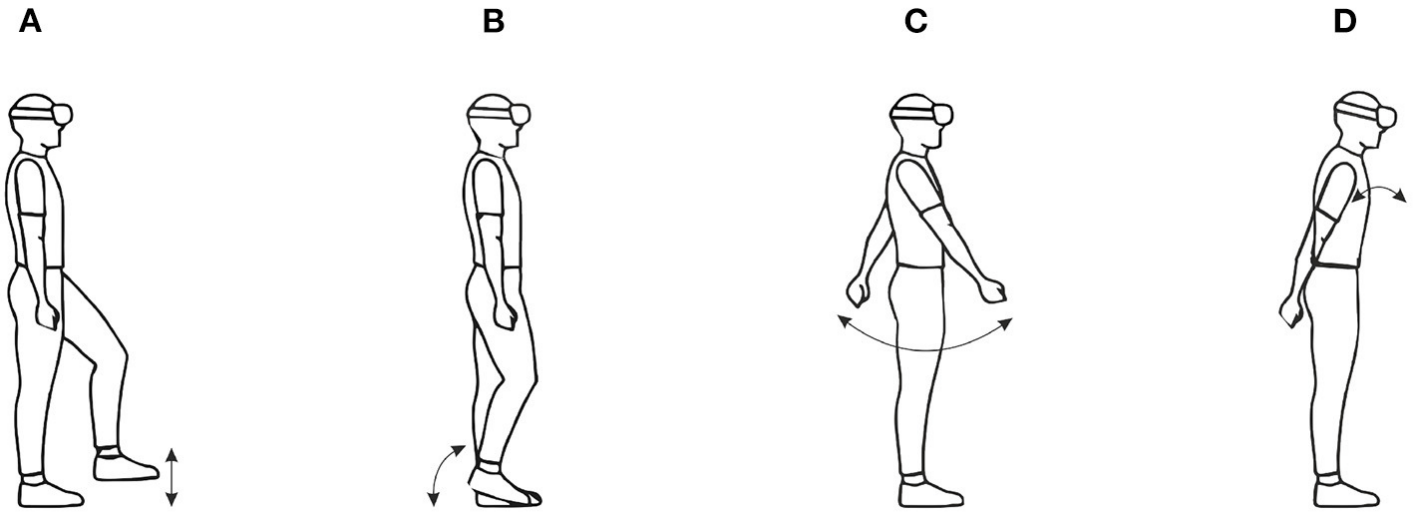
Images for WIP technique gestures: **(A)** traditional WIP, **(B)** tapping in place **(C)** arm swinging, and **(D)** forward leaning. Arrows illustrate the movement of body parts used to perform the gesture.

**Figure 5:**
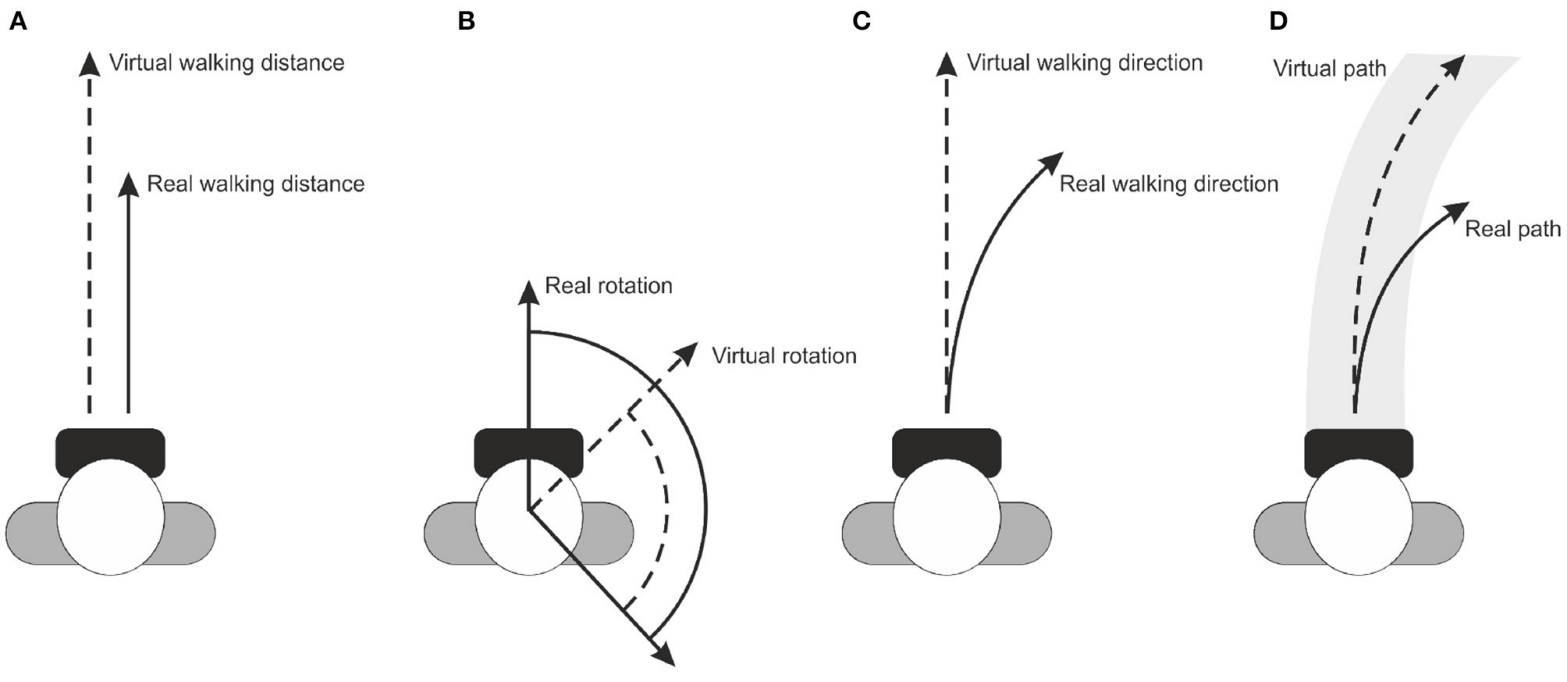
Images for RDW technique manipulations: **(A)** translation gain, **(B)** rotation gain **(C)** curvature gain, and **(D)** bending gain. Lines indicate the real and virtual transformations.

However, with RDW techniques large-scale VEs can be explored within a smaller tracking area. There are some variations of RDW techniques, and different taxonomies have been proposed. Steinicke et al. (2009b) proposed a classification based on the types of gains applied: translation (Figure 5A), rotation (Figure 5B) or curvature (Figure 5C). Suma et al. (2012) proposed a different classification based on the geometric flexibility, the detectability of the technique and the continuity. In this taxonomy, the repositioning and reorientation techniques can either be overt or subtle according to the detectability, and either continuous or discrete according to the gain application. Bruder et al. (2012) examined the limits of the gains for individuals using an electric wheelchair controlled by a joystick. The possible range for the gain values was found to be larger for such redirected driving. Recent work by Zhang et al. (2018) has examined motion detection thresholds in a large VE for the purposes of improving a 360° camera telepresence robot by real walking. They found that participants could not discriminate between real and telepresence movements (i.e., translation and rotation) when translation gains are down-scaled by 6% and up-scaled by 10%, and rotation gains are about 12% less or 9% more than the actual physical rotations. This indicates that observers in this particular setup were indeed sensitive to motion discrepancies.

Redirection algorithms can also be altered to involve passive haptic feedback objects (Steinicke et al., 2008, 2009a). A proxy object in the real environment representing virtual objects with similar size, shape and surface structure can support passive haptic feedback to the users. Although more difficult to utilize, such passive haptic feedback improve the VR experience significantly (Insko et al., 2001). Other RDW techniques use a visuo-haptic interaction to modify the human spatial perception, such as Suma et al. (2011) and Matsumoto et al. (2016), to provide a sensation of walking in unlimited VR space in spite of walking in a limited real space. In these systems, since the users actually move their bodies in space, both motor commands and proprioceptive as well as vestibular information from the body movements can be utilized. Another technique for exploring architectural 3D models scales the virtual room to fit into the real room, so that users can feel the real walls when they reach to the virtual walls (Bruder et al., 2009). In this study, an intense redirection was used to force users go through a virtual door in a virtual wall, so that they did not collide with the real walls.

Recently, novel RDW techniques consider perceptual masking effects like saccades, blinks, and other perceptual suppressions. In this context, Sun et al. (2018) enhance redirected interaction by detecting saccades and amplifying redirection during the events without introducing virtual scene warping. Another work by Langbehn et al. (2018) conducts perceptual experiments to measure translation and rotation thresholds during eye blinks to facilitate RDW.

### 3.3. Locomotion Devices

**Figure 6:**
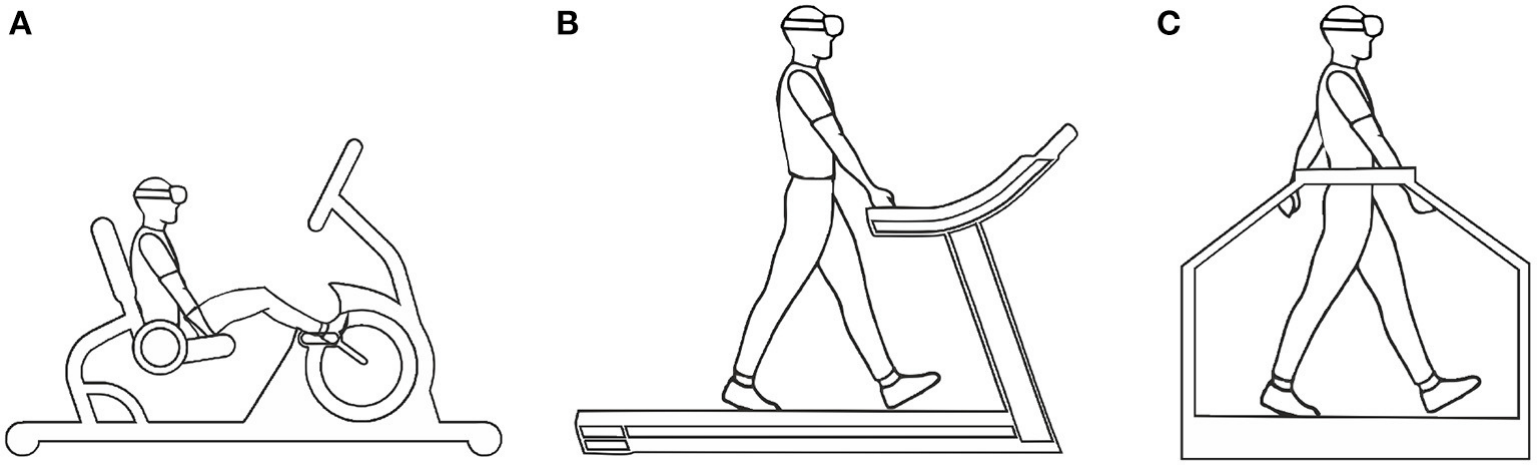
Images for treadmill locomotion interfaces: **(A)** seated, **(B)** unidirectional, and **(C)** omnidirectional treadmills.

Treadmills are allowing navigation of large-scale VEs via walking movements made within a limited space. While it is supposed to biomechanically identical to normal walking, it alters users' perception of motion due to missing vestibular feedback and alters the user's gait cycle (Durgin et al., 2007). Seminal work in this field was reported by the *Walkthrough* project (Brooks, 1987), which supported unidirectional movement, and the user could rotate by using a steering bar similar to a bicycle. It allows for walking in one direction, but severely restricting the possibilities for navigation through VEs (Souman et al., 2010). Three generations of locomotion devices were developed for the U.S. Army's Dismounted Infantry Training Program (Darken et al., 1997). The Uniport was the first seated treadmill (Figure 6A) built for lower body locomotion and exertion, which did not feel natural and did not allow for making sidesteps. The second, Treadport is based on a standard unidirectional treadmill (Figure 6B) with the user being monitored and constrained from behind via a mechanical attachment to the user's waist. It was better compared to the first generation in which allowed for more natural locomotion, but was still limited to one direction of movement. The third generation system was the omnidirectional treadmill (Figure 6C) that enables locomotion in any direction of travel. The system consisted of 2D rotary motors that moved the treadmill belts to keep the user in the same place. The study showed that accurate user tracking and precise control over the speed of the belts were critical for usability of the system. Otherwise users experienced uncomfortable sudden movements. A similar system was developed in later studies and compared a 3DOF motion platform with controller-based locomotion (Darken et al., 1997; Iwata, 1999). In more recent studies, an improved omnidirectional treadmill so-called *CyberWalk* was compared with real walking (Schwaiger et al., 2007; Souman et al., 2011), which allows for natural walking in any direction through arbitrarily large-scale VEs. The CyberWalk needed to ideally be large enough to accommodate a gradual accelerations on the motion platform to keep the user at its center. Although the system was found to be effective in locomotion in VEs, it is extremely expensive to maintain and difficult to adjust in the real space (Frissen et al., 2013).

Furthermore, there are some atypical approaches to locomotion in this category. One of these studies was so-called *Cybersphere* (Fernandes et al., 2003). The authors used a large sphere in which the user could walk, run, jump or crawl freely in any direction to explore an infinite VE. Another similar product, which was commercialized, is called *VirtuSphere* (Medina et al., 2008). The VirtuSphere was designed to work with HMDs that enables users to walk in all directions by placing them inside a large, rotatable, hollow sphere. Due to the sphere having its own large mass, it will not stop, start, or change directions with a high degree of responsiveness, and users must essentially re-calibrate their movements to adjust for the movement of the surface under their feet. Another interesting approach to locomotion in VEs called *String Walker* (Iwata et al., 2007). In this approach, each foot was attached to four motor pulleys with strings. Once a forward motion was detected, the strings pulled the user to the center. This information was gathered with a touch sensor placed on each foot. It detected stance phase and swing phase of walking. The tension was only applied when the foot was on the ground. The motor-pulley mechanisms are mounted on a turntable driven by a motor when the walker changes direction of walking, the turntable is activated to follow the direction of the walker.

### 3.4. Controller-Based Virtual Walking

Manual devices such as joysticks, keyboards and VR controllers are widely available, which allow to perform walking inside the VE by involve user's hands and arms (Darken and Sibert, 1996; Marchal et al., 2011). Such joystick-based walking was compared with real walking using different display types (CAVE vs. HMD) (Grechkin et al., 2014). In this study, users performed perceptual-motor coordination tasks with different locomotion techniques. The results show that different velocity controls of each locomotion technique affect the timing and success rate of actions. In real walking, the speed can be controlled easily whereas with a joystick an almost constant speed is provided. Another study by Peck et al. (2011b) and Peck et al. (2011a) compared joysticks with other locomotion techniques in a virtual maze environment. They found that participants, who used joystick-based walking performed significantly worse than participants who used RDW or WIP. Furthermore, joystick- and keyboard-like devices were inferior for controlling spatial orientation compared to RDW techniques (Ruddle and Lessels, 2006). Riecke et al. (2010) compared real walking and joystick locomotion with an additional alternative of real rotation with joystick-based walking. They found that combining real rotation with joystick-based walking produce similar task performance scores as real walking. The results show that large tracked areas are not required for reasonable navigation performance in VR. On the other hand, Nabiyouni et al. (2015) compared joystick to a real walking and VirtuSphere; joystick received better results than VirtuSphere in terms of fatigue, ease of learning, ease of walking and precision. The authors concluded that well designed low fidelity locomotion techniques such as joysticks often give better results compared to designs with moderate interaction fidelity like VirtuSphere.

Alternative locomotion techniques have been developed using VR controllers such as teleportation (Bozgeyikli et al., 2016). With teleportation the user's virtual viewpoint is moved while the user itself stays at the same position and orientation in the physical space. Bolte et al. (2011) developed the so-called *jumper metaphor* that uses the head direction to select the destination and a physical jump of the user to trigger the teleportation. Another work by Bozgeyikli et al. (2016), utilizes gesture-based interaction to point to where the user wants to go, and the main motion takes place through teleportation. In their work, teleportation was compared to WIP and joystick regarding usability. Results show that teleportation is subjectively preferred as a user friendly locomotion technique. However, an extended version of this teleportation technique for which it was possible to set a certain target direction into which the user should face after the teleportation, showed a decrease of the user experience. Bolte et al. (2011) compared teleportation to real walking and to the jumper metaphor. The result shows that teleportation and jumper metaphor are more effective techniques than real walking. Furthermore, in a CAVE setup, Freitag et al. (2014) compared teleportation to joystick and real walking with portals that were used to reorient the user in the tracking space. Teleportation was faster than real walking, but led to an increased loss of orientation compared to joystick. They could not find any differences between teleportation and real walking concerning motion sickness.

Overall virtual walking techniques may be a practical and useful tool to target sensory and cognitive deficits that contribute to gait impairments, and thus provide new opportunities to improve gait, mobility, and ultimately quality of life in those living with neurological and neurodegenerative diseases. In the following section we will discuss research utilizing virtual reality as a method for therapeutic intervention for gait impairments in different cohorts of neurological patients.

## 4. Virtual Walking Techniques for Gait Rehabilitation

More recent reviews by Canning et al. (2020), Huygelier et al. (2021), and Keshner and Lamontagne (2021) highlighted the concrete contributions of VR to rehabilitation of balance and gait; suggesting that the most promising effects of VR are the ability to multitask in a VE that can replicate the demands of a physical space. There is indeed already a promising body of evidence for effective virtual walking techniques in populations such as stroke (Mirelman et al., 2010; Cai et al., 2021), multiple sclerosis (Samaraweera et al., 2013; Winter et al., 2021), Parkinson's disease (Janeh et al., 2019a; Quek et al., 2021), and Alzheimer's disease (White and Moussavi, 2016). In this section, we summarize the different VR-based gait rehabilitation approaches:

### 4.1. Treadmills

A commonly used virtual walking technique for gait rehabilitation is unidirectional treadmills (Yang et al., 2008; Mirelman et al., 2011; Peruzzi et al., 2017; Richards et al., 2018). Such non-immersive VR-based training hold promise for fully immersive VR, such as using an HMD in combination with treadmill walking (Luque-Moreno et al., 2015; Roeles et al., 2018), which provided motor cognitive challenges in a simulated, real life but safe environment, compared with the same dose of treadmill training alone (Canning et al., 2020). To date, a small number of studies have investigated gait training using an HMD (Parijat et al., 2015; Peterson et al., 2018; Chan et al., 2019), and a recent study showed that both young and older adults were able to use HMD during walking without adverse effects (Kim et al., 2017). More recently, research groups have also investigated the use of omnidirectional treadmills to walk through virtual environments (Lamontagne et al., 2019; Soni and Lamontagne, 2020), which allow changes in direction while accommodating gait speed changes that observed during overground locomotion.

In parallel to those clinical investigations, other studies have demonstrated VR foot pedals combined with neuroimaging techniques (functional MRI) or DBS surgery to investigate the pathophysiology underlying gait deficits in Parkinson's disease with freezing of gait; which in turn allowed the patients to navigate forward or turning through the virtual environment (Shine et al., 2013; van der Hoorn et al., 2014; Gilat et al., 2015; Georgiades et al., 2016; Ehgoetz Martens et al., 2018; Matar et al., 2019). Forward progression was only achieved when patients alternately depressed the pedals (i.e., left-right-left). Along the same lines, several studies already adopted a VR cycling training for the motor rehabilitation of old adults or stroke patients (Deutsch et al., 2013; Yin et al., 2016; Pedroli et al., 2018). Although, it seems that treadmills walking may lead to similar kinematic data to ground walking, but further studies will be necessary to ensure that the acquired skills from VE practice can be transferred to the real world (Lohse et al., 2014; de Rooij et al., 2016; Palma et al., 2017; Porras et al., 2018; Levac et al., 2019).

### 4.2. Virtual Stepping

Among the most promising one that requires bilateral limb coordination, Killane et al. (2015) investigated the effects of the addition of a non-immersive VR component to stepping in place on a balance board with cognitive loading aimed at reducing the number of FoG episodes in PD. These technologies, which allow stepping-in-place on a balance board, have been utilized previously in literature to mimic gait (Nantel et al., 2011). Accordingly, a virtual teacher has effectively instructed while healthy adults were stepping in place (Koritnik et al., 2008, 2010), and others have successfully been applied in rehabilitation (Duschau-Wicke et al., 2009). More recently, there has been an emphasis on using stepping over virtual obstacles placed on the path of walking, either projected onto the floor (Geerse et al., 2018, 2020b) or treadmill (Heeren et al., 2013; van Ooijen et al., 2016) or 3D holographic cues seen through Microsoft HoloLens (Coolen et al., 2020; Geerse et al., 2020a; Miyake et al., 2021), or displayed on the floor of a virtual environment (Gómez-Jordana et al., 2018; Janeh et al., 2019a), which can be used for advance planning and real-time modification of the obstacle avoidance behavior (Edd et al., 2020). As such, the possible applications for this gait retraining paradigm are widespread, especially when combined with measures of gait biomechanics alterations (Sveistrup, 2004; Martens et al., 2014; Cano Porras et al., 2019). It has been previously shown that individuals are able to follow floor-projected foot placement visual cues aimed to modify gait parameters with an accuracy that is sufficient for the most common therapeutic applications (Bennour et al., 2018). Therefore, VR has the potential to present novel classes of stimuli, such as virtual humans and avatars that provide continuous information (Kiefer et al., 2013). It is therefore possible to imagine a number of ways that continuous information about the desired gait pattern could be presented to a patient. Liu et al. (2020) leverage embodiment in a virtual environment to help with rehabilitation from gait asymmetry, allowing the patient to see their own gait. Moreover, studies have shown that WIP is feasible on gait rehabilitation of stroke patients in which intensity, frequency, motion amplitude, and feedback can be manipulated to provide tailored motor training (Cai et al., 2021). Future studies might focus on identifying which control strategies can best facilitate stepping performance in patients at varying degrees of recovery following neurological injury.

### 4.3. Virtual Manipulations

One of the unique capabilities of VR is that visual information can be enhanced or manipulated during ongoing walking in a manner that is not possible in the real world, e.g., VEs can be used to manipulate visual cues to modulate the gait characteristics of patients with PD that provoke FoG and other impairments contributing to fall risk (Schubert et al., 2005). For instance, Janeh et al. (2019a) found that PD patients overcame the spatial asymmetry and exhibited a comparable step length by enlarging the step length of the short side, an adapted step time, and a swing time variability of both sides during the manipulation of visual-proprioceptive cues. Another example is by Barton et al. (2014) that has investigated the possibility of using the manipulation of visual cues with a time delay in a VE to alter gait using a Virtual Mirror Box. In their study, movements kinematics of the unimpaired leg were combined with the movement timing of the impaired leg to model a realistic avatar with a symmetric gait pattern. In addition, an extensive body of literature has examined the role of visual self-motion in the control of locomotion by selectively manipulating the direction or speed of the visual flow provided through the VE (Lamontagne et al., 2007, 2010). VR can also be used to manipulate the locomotor trajectory of patients during overground walking that varied the path's radius of curvature, to assess the impact of an emulated knee disability on the locomotor trajectory. Gérin-Lajoie et al. (2010). In many studies (Chou et al., 2009; Janeh et al., 2017a,b, 2018), where they manipulated the translation gain of walking in healthy younger and older adults, so that one step forward in the physical world corresponds to several steps forward in the VE. In contrast, Matsumoto et al. (2018) examined the effect of curvature and bending gains (Figure 5d) on walking biomechanics, which occurs when the curvature of the walking path in the VE was manipulated, while the actual walking path remains constant. Therefore, using VR to manipulate visual flow thus has the potential to alter the interaction space and provide notable information about locomotion speed and heading to the patient (Warren et al., 2001; Turano et al., 2005). Walking trajectory was shown to be affected when healthy young subjects were exposed to rotational, translational or a combination of both, demonstrating the importance of visual flow on steering behavior during locomotion (Sarre et al., 2008). Additionally, if the same rotational optic flow is generated via a simulated camera rotation in VE against an actual head rotation, a different locomotor behavior also emerges, whereby the simulated but not the actual head rotation results in a trajectory deviation (Hanna et al., 2017). Such findings support the potential contribution of the motor command in heading estimation (Banks et al., 1996; Crowell et al., 1998). These findings also corroborate the presence of multisensory integration of both visual and non-visual information (i.e., vestibular, proprioceptive, and somatosensory) to generate a single representation of self-motion and orientation in space (Karthik et al., 2014; Acerbi et al., 2018).

### 4.4. Controllers

Another technique was also employed using controller-based virtual walking, where participants were asked to walk around a VE and remembering objects and rooms that they had viewed in order to estimate cognition (Albani et al., 2002; Klinger et al., 2006; Cipresso et al., 2014). The authors have focused on motor control aspects related to action and navigation as well as performing activities of daily living (i.e., even though they were not actually walking). However, this basic research has implications for practice; suggesting that VEs can be used for the examination of cognitive deficits that may interfere with mobility. Moreover, the use of VR hand-held controllers allows users to interact with virtual elements using their hands as they do real-life, allowing exercise repetition, intensity variation, and task-oriented training (Cortés-Pérez et al., 2020). Although these studies have great potential in improving the assessment of cognition in a more ecological manner, more research studies are needed to know whether this will be useful, reliable, and clinically meaningful. Once this is established it would be useful to use these cognitive tasks to assess and quantify changes in gait in order to understand gait disorders.

## 5. Conclusion

As such, this review emphasized the importance of employing virtual walking techniques in rehabilitation, and thereby it is a promising approach and possibly effective for improving the gait of people with neurological diseases, suggesting that the severity of the disease can influence the effect of the use of VR during rehabilitation. Moreover, to determine the role of VR-based gait rehabilitation, further research is needed to investigate the characteristics of each patient and his disorder to develop personalized techniques. Thus, potential changes in gait characteristics should be taken into consideration when designing virtual walking techniques.

## Author Contributions

All authors listed have made a substantial, direct, and intellectual contribution to the work, and approved it for publication.

## Conflict of Interest

The authors declare that the research was conducted in the absence of any commercial or financial relationships that could be construed as a potential conflict of interest.

## Publisher's Note

All claims expressed in this article are solely those of the authors and do not necessarily represent those of their affiliated organizations, or those of the publisher, the editors and the reviewers. Any product that may be evaluated in this article, or claim that may be made by its manufacturer, is not guaranteed or endorsed by the publisher.
